# Cynaropicrin Shows Antitumor Progression Potential in Colorectal Cancer Through Mediation of the LIFR/STATs Axis

**DOI:** 10.3389/fcell.2020.605184

**Published:** 2021-01-11

**Authors:** Dandan Zheng, Yu Zhu, Yili Shen, Sisi Xiao, Lehe Yang, Youqun Xiang, Xuanxuan Dai, Wanle Hu, Bin Zhou, Zhiguo Liu, Haiyang Zhao, Chengguang Zhao, Xiaoying Huang, Liangxing Wang

**Affiliations:** ^1^The First Affiliated Hospital, Wenzhou Medical University, Wenzhou, China; ^2^School of Pharmaceutical Sciences, Wenzhou Medical University, Wenzhou, China; ^3^The Institute of Life Sciences, Wenzhou University, Wenzhou, China; ^4^The Second Affiliated Hospital and Yuying Children’s Hospital, Wenzhou Medical University, Wenzhou, China

**Keywords:** cynaropicrin, CRC, STATs, LIFR, inhibitor

## Abstract

**Background:**

Colorectal cancer (CRC) is the second deadliest malignant disease in the world and the leukemia inhibitory factor receptor/signal transducers and activators of transcriptions (LIFR/STATs) signaling axis plays an important role in the molecular biology of CRC.

**Methods:**

Cell function tests were performed to observe the inhibitory effect of cynaropicrin on human CRC cells (RKO, HCT116, and DLD-1). Expression levels of LIFR, P-STAT3, P-STAT4, and apoptotic proteins were detected by Western blotting. Immunoprecipitation confirmed the presence of LIFR/STAT3/STAT4 complex. Cell immunofluorescence assay was used to observe the subcellular localization of STAT3 and STAT4. *In vivo* efficacy of cynaropicrin was evaluated by a xenotransplantation model in nude mice.

**Results:**

Cynaropicrin significantly reduced the survival ability of human CRC cells and promoted apoptosis in a dose-dependent manner. Western blotting results suggested that the antitumor effects of cynaropicrin might be mediated by inhibition of the LIFR/STATs axis. Cynaropicrin reduced the formation of STAT3/STAT4 heterodimers and blocked their entry into the nucleus. Cynaropicrin also suppressed tumor growth in the xenograft model.

**Conclusion:**

The results showed that cynaropicrin exerted a strong inhibitory effect on CRC *in vitro* and *in vivo*. Our study concluded that cynaropicrin has potential application prospects in the field of anti-CRC therapy.

## Introduction

Colorectal cancer (CRC), one of the most prevalent malignant diseases, ranks second among all cancers in terms of mortality and third in terms of incidence worldwide (Bray et al., 2018). There are projected to be 147,950 individuals newly diagnosed with CRC and an estimated 53,200 CRC deaths in the United States in 2020 (Siegel et al., 2020). In the Chinese population, CRC was one of the most common causes of cancer-related deaths in males (8.0%) and females (9.8%) (Feng et al., 2019). Therefore, in addition to surgery, there is an urgent need to identify promising molecular drugs with high efficacy and low toxicity.

Leukemia inhibitory factor (LIF) is a member of the interleukin-6 (IL-6) family and has the most multi-potent action. LIF binds to leukemia inhibitory factor receptor (LIFR) with high affinity (Boulanger et al., 2003), activating the Janus Kinase (JAK) family of tyrosine kinases, particularly JAK1 (Ernst et al., 1996). JAK1 activates a series of tyrosine phosphorylation events and stimulates three signaling pathways, including JAK/signal transducers and activators of transcription (STATs) (Stahl et al., 1994). Signal STAT3, the only STAT family member whose genetic deletion results in embryonic lethality (Takeda et al., 1997), has been estimated to be abnormally activated in more than 70% of human cancers (Frank, 2007). Persistent STAT3 activation is involved in tumorigenesis, proliferation, anti-apoptosis, and metastasis (Zhao et al., 2016). STAT4, also a member of the STAT family, is a key mediator of the pro-inflammatory immune responses. High expression of STAT4 has been shown to be a positive prognostic factor in liver cancer, breast cancer, and ovarian cancer (Verhoeven et al., 2020), whereas its overexpression in CRC is positively correlated with the depth of tumor invasion (Cheng et al., 2015). STAT3 is considered to be the most important signal transducer of LIF stimulation. It has been shown that LIF cytokine stimulation induces LIFR to recruit the STAT protein (Auernhammer and Melmed, 2000). Further, the STAT protein is activated to form a signal-enhanced dimer that enters the nucleus and upregulates the transcription of the corresponding cytokine response genes (Zhao et al., 2020).

Cynaropicrin (Cyn), a sesquiterpene lactone (STL), is a major biologically significant class of secondary metabolites in the artichoke (Menin et al., 2012). Cynaropicrin has been shown to possess various pharmacologic properties, such as anti-hepatitis C virus (Elsebai et al., 2016a), anti-parasitic (Zimmermann et al., 2014), anti-photo aging (Takei et al., 2015), anti-inflammatory (Cho et al., 2000), and anti-tumor properties. In human leukemia and prostate cancer cells, cynaropicrin is able to induce glutathione depletion and result in *S*-glutathionylation of STAT3, leading to down-regulation of STAT3-dependent gene expression and chemosensitization of tumor cells to chemotherapy (Butturini et al., 2013). Cynaropicrin impinges on the thioredoxin (Trx) system and leads to Trx oxidation and reactive oxygen species (ROS) accumulation in cells, thereby inducing apoptosis of Hela cells (Liu et al., 2019). Cynaropicrin may serve as a potential cancer targeted drug for prevention or treatment of human cancers.

Inactivation of STAT3 is a promising anticancer strategy, but an STAT3 inhibitor has not yet been approved for the market. As a potential natural product targeting the STAT3-related signaling pathway, the application of cynaropicrin in CRC may be worth exploring. Therefore, we assessed the inhibitory effect of cynaropicrin on CRC at the cellular and animal levels. In this study, we further explored the specific anti-cancer mechanism of cynaropicrin to determine if it has potential application prospects in the field of antineoplastic therapy.

## Materials and Methods

### Cell Culture and Reagents

Human CRC cell lines (HCT116, RKO, and DLD-1) were obtained from the cell resources center of the Shanghai Institutes for Biological Sciences (Chinese Academy of Sciences, Shanghai, China). HCT116 cells were grown in McCoy’s 5A medium (Gibco, NY, United States) and DLD-1 cells were cultured in Dulbecco’s Modified Eagle’s Medium (DMEM) (Thermo Fisher Scientific, Waltham, MA, United States). RKO cells were cultured in RPMI-1640 media (Thermo Fisher Scientific, Waltham, MA, United States). The above-mentioned basic culture media were supplemented with 10% fetal bovine serum (FBS) (Gibco, NY, United States) and 1% of Penicillin-Streptomycin (10,000 U/mL) (Thermo Fisher Scientific, Waltham, MA, United States) and incubated at 37°C with 5% CO_2_. Cynaropicrin was purchased from Baoji Herbest Bio-Tech Co., Ltd. (CAS#: 35730-78-0). Recombinant human IL-6 was purchased from Bio-Techne China Co., Ltd. (206-IL-010).

### MTT Assay

Cells were seeded into wells of a 96-well plate (3 × 10^3^ cells/well) with 100 μL of the corresponding medium and allowed to attach overnight. Cynaropicrin was dissolved in DMSO to a certain concentration using gradient dilution. After being incubated with cynaropicrin for 48 h, the cells were treated with 25 μL/well MTT solution (5 mg/mL) for 4 h at 37°C. The formazan crystals were dissolved in 150 μL DMSO and the optical density (OD) was measured using a Microplate Reader at 490 nm. Half-maximal inhibitory concentration (IC50) values were determined by GraphPad Prism 7.0.

### Colony Formation Assay

The cells were seeded into a 6-well plate (800 cells/well) and incubated overnight. After treatment with drugs or DMSO for 2–6 h, the culture medium was replaced with fresh medium to keep the cells growing for one-week. Colonies were fixed with 4% paraformaldehyde for 15 min at room temperature and then stained with 1% crystal violet for 10 min at room temperature. After staining, the plates were washed with phosphate-buffered saline (PBS) and dried.

### Assessment of Cell Apoptosis by Flow Cytometry

Apoptosis was detected by FITC Annexin V Apoptosis Detection Kit I (BD Pharmingen^TM^, United States). In brief, cells inoculated in a 6-well plate were collected after being treated with DMSO or drugs for 24 h. Then they were resuspended in 500 μL binding buffer according to the instructions of the apoptosis kit. The treated cells (as described above) were successively incubated with fluorescein-labeled Annexin V and propidium iodide (PI). Apoptosis assessment was performed by FACSCalibur (BD Biosciences, MD, United States). Data were analyzed using Flowjo software.

### Cell Migration Assay

Cell migration assays were performed using a transwell filter (BD Biosciences, United States) according to the manufacturer’s instructions. Cells were seeded in the upper chamber containing a non-coated membrane. Culture medium containing 10% FBS was added to the lower chambers, and the cells were plated in the upper chamber with FBS-free medium. Both media were treated with DMSO or drugs. After 48 h, the cells were fixed with 4% paraformaldehyde and non-migrated cells were removed from the upper surface of the filter. The cells on the lower surface of the membrane were stained with 1% crystal violet for 10 min. The number of migrated cells was visualized and counted under an optical microscope.

### Immunoprecipitation and Western Blotting

Cells were lysed in protein lysis buffer and centrifuged to obtain the supernatant. The cells were lysed in the protein lysis buffer and the supernatant was obtained by centrifugation. The total protein in the lysate can be directly tested by Western blotting, or incubated with the corresponding antibody at 4°C overnight, and then adsorbed with agarose beads to separate the target protein for subsequent tests. Proteins were separated by 10 or 12% SDS-PAGE and then transferred onto a PVDF membrane. The blots were blocked for 2 h with fresh 5% non-fat milk at room temperature, followed by incubation with specific primary antibodies overnight at 4°C. After washing, membranes were incubated with the relevant secondary antibodies. Antibody staining was visualized using Omni-ECL Femto Light Chemiluminescence Kit (EpiZyme, Shanghai, China). Then, the images were analyzed by the Image J computer software. The following primary antibodies were commercially obtained: anti-GAPDH (AB-P-R 001, GoodHere Technology), anti-STAT3 (phospho Y705) (ab76315, Abcam), STAT3 mAb (#12640, Cell Signaling Technology), Phospho-STAT4 (Tyr693) mAb (#4134, Cell Signaling Technology), STAT4 mAb (#2653, Cell Signaling Technology), anti-LIFR (sc-515337, Santa Cruz, CA, United States), anti-LIF (sc-515931, Santa Cruz, CA, United States), anti-Bax (ab32503, Abcam), anti-Bcl-2 (sc-56015, Santa Cruz, CA, United States), and Anti-Cleaved PARP1 (ab32064, Abcam).

### Immunofluorescence

The cells seeded in glass bottom cell culture dish treated with cynaropicrin and/or IL-6 were fixed with 4% paraformaldehyde and then permeabilized in 0.1% Triton X-100. The fixed CRC cells were blocked with 1% bovine serum albumin (BSA) for 1 h at room temperature. Blocked cells were incubated with the specific primary antibody of STAT3 (1:500 in 1% BSA) or STAT4 (1:1,600 in 1% BSA) overnight at 4°C. After rewashing in PBS, the cells were allowed to react with Goat Anti-Rabbit IgG (Alexa Fluor^®^ 488) (ab150077, Abcam) (1:700 in 1% BSA) for 1 h in the dark and counterstained with DAPI for 10 min. The images of STAT3/STAT4 and DAPI stained cells were observed under a Leica SP5 confocal microscope.

### Surface Plasmon Resonance Studies

The equilibrium-binding constant (KD) of LIFR Protein and Cynaropicrin was determined by surface plasmon resonance (SPR) (Nicoya, Canada). All the steps were performed according to previously described protocol Briefly, the LIFR Protein (0.15 mg/ml) was immobilized on NTA-sensor chips (Nicoya, Canada). Then, all the cynaropicrin were continuously diluted into several different concentrations using the running buffer and injected into the chip from low to high concentrations (0, 200, 400, and 800 μM). Meanwhile, PBS containing 2% DMSO was used as a negative control. In each cycle, a 200 μl sample was flowed through the chip at a constant flow rate of 20 μl/min. The binding time of protein and ligand was 240 s. Naturally dissociate 480 s. Finally, the kinetic parameters of the binding reactions were calculated and analyzed by Trace Drawer software (Ridgeview Instruments AB, Sweden).

### Xenograft Models

All animal experiments were conducted using protocols approved by The Wenzhou Medical University Animal Policy and Welfare Committee. All mice were purchased and raised uniformly by the Animal Experiment Center of Wenzhou Medical University. HCT116 cells mixed with an equal volume of PBS and matrigel were implanted in the hind flank of mice (nude mice, female, 5–6 weeks old). Upon attaining an appropriate tumor volume (approximately one-week post-implantation), 30 mice were randomized into 4 groups and intraperitoneally injected with Napabucasin (NAPA) or cynaropicrin (8 mice in the control group, 7 mice in the NAPA/Cyn low (2.5 mg/kg)/Cyn high (5.0 mg/kg) group, and 1 mice without tumor formation). The mice were given the drug every 2 days and their weight was weighed. The diameters of surface transplanted tumors in mice were measured with calipers, usually with the longest diameter as the length (L) and the diameter perpendicular to the longest diameter as the width (W). Tumor volume was measured as *V* = (L × W × W)/2. Animals were executed in anesthesia with CO_2_ and sacrificed at the end of study. Their corpses were unified with harmless treatment. The tumors, hearts, livers, kidneys and lungs were removed and preserved in 4% paraformaldehyde for further use (histological and protein expression analyses).

### Statistical Analysis

The experimental results were expressed as the mean ± SDs of three parallel experiments. The differences in data among groups were analyzed by unpaired two-tailed Student’s *t*-tests in GraphPad Prism 7. *P*-values less than 0.05 were considered statistically significant.

## Results

### Cynaropicrin Affected the Cell Viability of CRC

**FIGURE 1:**
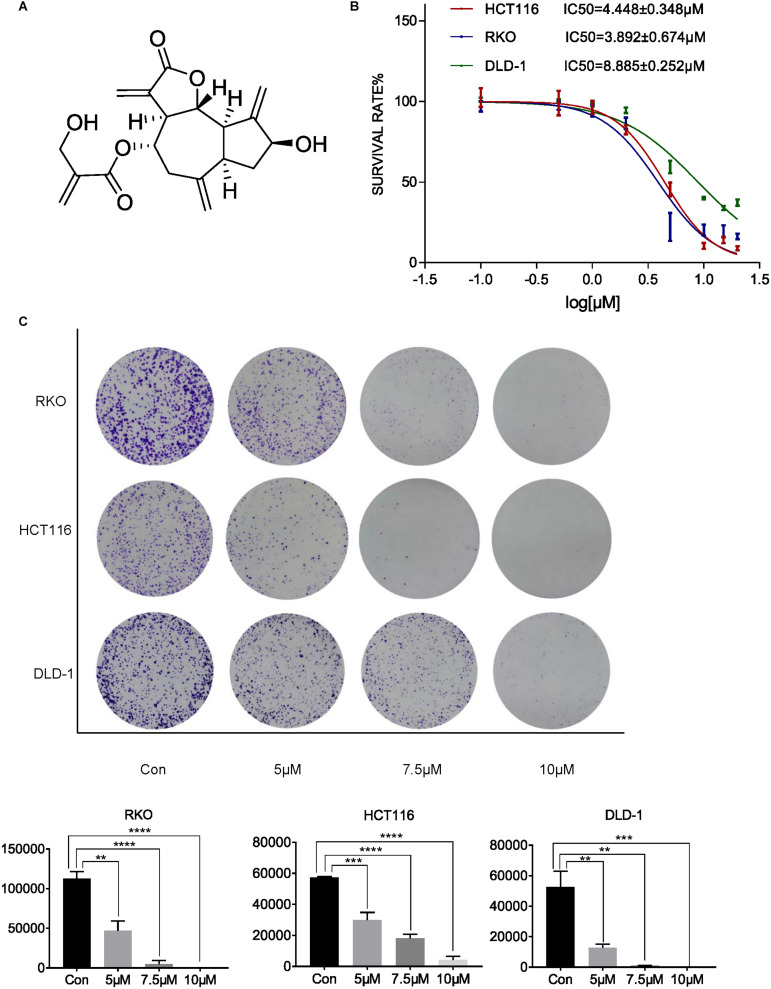
Cynaropicrin inhibited CRC cell proliferation and colony formation. **(A)** Chemical structure of cynaropicrin. **(B)** The inhibition ratio was calculated by MTT assay in HCT116, RKO, DLD-1 cells. **(C)** Colony formation assay was performed using the above-mentioned three cells treated with or without cynaropicrin. All potency values are expressed as mean ± SD of independent experiments in triplicate. Definition of statistical significance: *P* < 0.05.

To investigate the inhibitory effect of cynaropicrin-inhibited proliferation in human CRC cell lines, cell viability was evaluated (Figure 1A). The results of MTT assay showed that cynaropicrin exhibited promising growth inhibition in HCT116, RKO, and DLD-1 cells in a dose-dependent manner. The corresponding IC50 values were 4.45, 3.89, and 8.88 μM, respectively (Figure 1B). Furthermore, the colony formation assay showed that cynaropicrin can significantly suppress the colony formation ability of these three cell lines as shown in Figure 1C. Together, these data suggested that cynaropicrin can effectively inhibit the growth and proliferation of human CRC cells.

### Induction of the Apoptosis Effect of Cynaropicrin in CRC Cells

**FIGURE 2:**
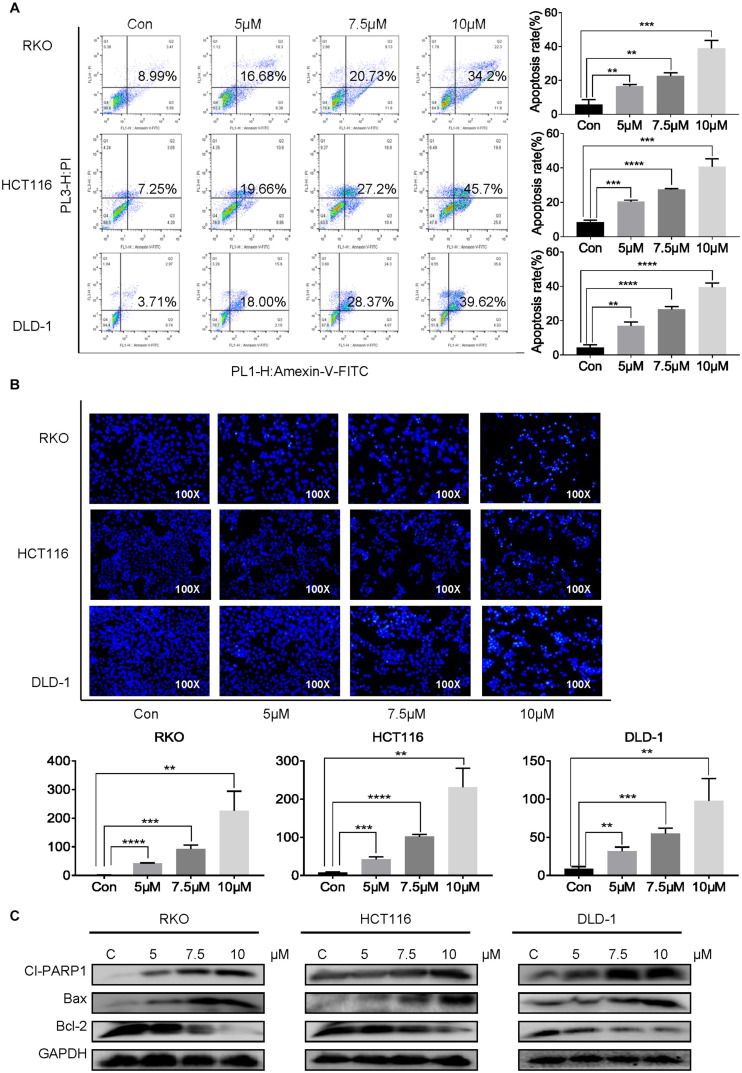
Cynaropicrin-induced cell apoptosis in CRC cells. **(A)** Flow cytometric analysis and **(B)** Hoechst 33258 staining were performed to analyze the cell apoptotic rate after treatment with 0, 5, 7.5, and 10 μM cynaropicrin for 24 h in HCT116, RKO, and DLD-1 cells. **(C)** The expression levels of Cl-PARP1, Bax and Bcl-2 in three cells were measured by Western blotting after incubation with cynaropicrin at three concentrations for 24 h. All potency values are expressed as mean ± SD of independent experiments in triplicate. Definition of statistical significance: *P* < 0.05.

To evaluate whether cynaropicrin can induce cell apoptosis, three cell lines were treated with cynaropicrin at three different concentrations for 24 h, stained with Annexin V FITC and PI, and the percentage of apoptotic cells was detected by flow cytometry. Remarkably, cynaropicrin significantly induced CRC cells apoptosis (Figure 2A). Similar results were provided by Hoechst 33258 staining, which further confirmed the above observations. Cells treated with cynaropicrin displayed strong blue fluorescence and demonstrated significant apoptotic patterns (Figure 2B). Here, we also found changes in the expression levels of Cl-PARP1, Bcl-2, Bax, which are associated with the process of apoptosis (Figure 2C).

### Cynaropicrin-Induced Loss of Migration Potential in CRC Cells

**FIGURE 3:**
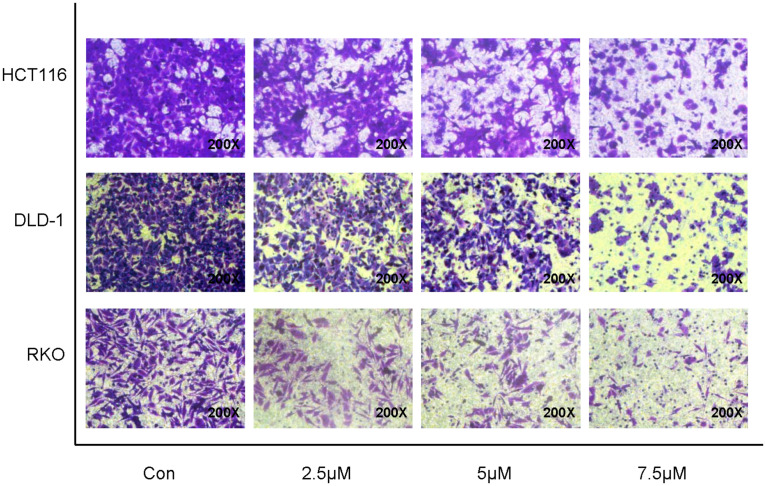
Cynaropicrin induced loss of migration potential in CRC cells. Transwell migration assay was performed in HCT116, DLD-1 and RKO cells.

To determine whether treatment with cynaropicrin was associated with tumor cell migration, changes in migration potential were measured using transwell assays. As shown in Figure 3, compared with the control group cynaropicrin significantly suppressed the cell migration capability. The number of cells passing through the transwell filtration membrane was significantly reduced after treatment with gradually increasing concentrations of cynaropicrin. Therefore, we concluded that cynaropicrin inhibited migration of the CRC cell lines in a concentration-dependent manner.

### Cynaropicrin Blocked the Activation of STAT3 *via* Inhibiting LIFR Activity

**FIGURE 4:**
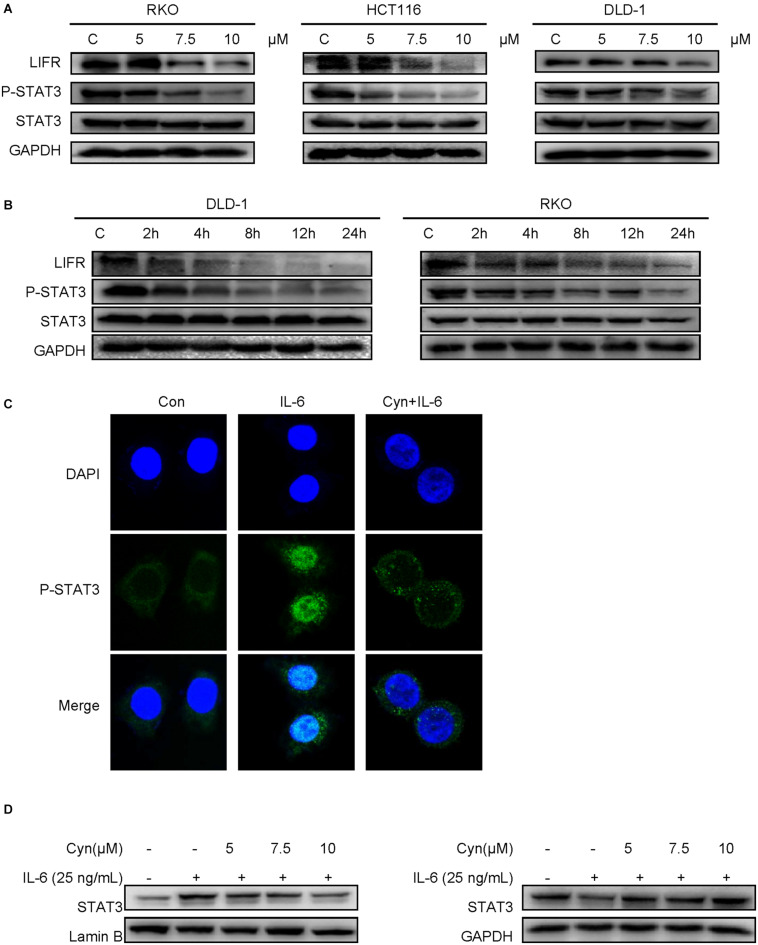
Cynaropicrin inhibited the LIFR/STAT3 signaling pathway. **(A,B)** DLD-1 and RKO cells were treated with cynaropicrin at different concentrations (0, 5, 7.5, and 10 μM) or for different lengths of time (0, 2, 4, 8, 12, and 24 h). Western blotting was used to detect the protein expression levels of LIF/LIFR, and P-STAT3/STAT3. RKO cells were pretreated for 24 h and then stimulated with IL-6 (25 ng/mL) for 30 min. **(C)** Subcellular localization of STAT3 was detected by immunofluorescence staining and laser confocal microscopy in RKO cells. **(D)** The expression of STAT3 protein in cytoplasm and nucleus was detected by Western blotting after extraction with a kit in RKO cells.

In consideration of the important role played by the STAT3 pathway in CRC, we attempted to determine whether cynaropicrin can inhibit the phosphorylation and activation of STAT3 in CRC cells. *Via* detecting the protein expression level by Western blotting, it was found that treatment with cynaropicrin obviously controlled the level of phosphorylate-STAT3 in HCT116, RKO, and DLD-1 cells in a time- and dose-dependent manner (Figures 4A,B). These findings proved that cynaropicrin induced CRC cell death mainly through the STAT3 pathway. To further investigate the mechanisms of cynaropicrin-induced cell apoptosis, we assessed subcellular localization of the STAT3 protein. As shown in Figure 4C, cynaropicrin significantly inhibited IL6-induced STAT3 nucleation. The separation of the nucleoprotein and the plasma protein also verified that cynaropicrin blocked STAT3 translocation into the nucleus (Figure 4D). The above results strongly indicated that cynaropicrin inhibited STAT3 phosphorylation and its translocation into the nucleus.

**FIGURE 5:**
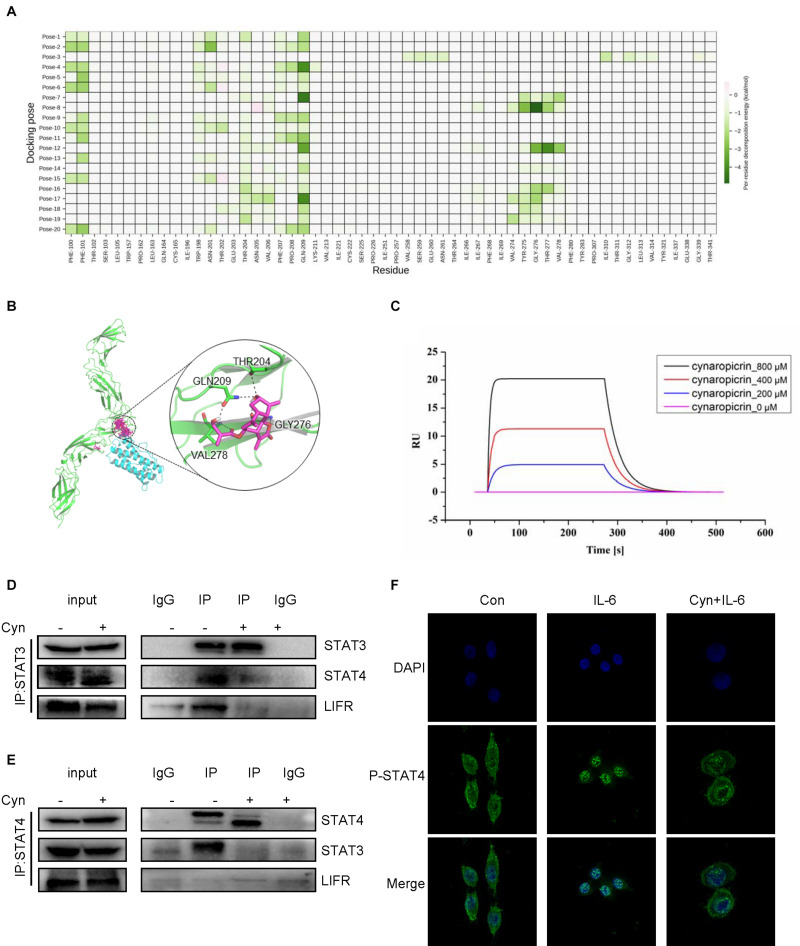
Cynaropicrin stimulation inhibits the formation of STAT3/STAT4 heterodimers activated by LIFR. **(A,B)** The cynaropicrin molecular docking at the contact interface between LIFR and LIF resulted in 20 conformations. Energy decomposition analysis based on residues suggests that GLN209 may be the key residue to maintain binding. **(C)** Confirmation of LIFR- Cynaropicrin interaction. LIFR Protein captured on NTA chip can bind Cynaropicrin, with an affinity constant of 134 μM as determined in a SPR assay. **(D,E)** RKO cells were treated with (+) or without (-)cynaropicrin for 24 h, and then total cell protein was extracted to perform a co-immunoprecipitation assay using STAT3 antibody or STAT4 antibody. The immunoprecipitation complexes were further separated by Western blotting. **(F)** RKO cells were pretreated for 24 h and then stimulated with IL-6 (25 ng/mL) for 30 min. Subcellular localization of STAT4 was detected by immunofluorescence staining and laser confocal microscopy.

Previous studies have demonstrated a strong role of STAT3 in LIF-signaling among several cell types (Nicola and Babon, 2015). LIFR and GP130 tyrosine residues provide SH2 domains of the STAT proteins with specific docking sites, resulting in subsequent STAT phosphorylation (Auernhammer and Melmed, 2000). The results of computer-aided molecular docking showed that cynaropicrin could directly bind to the amino acid residues at the LIF/LIFR contact interface, thereby inhibiting LIF binding to LIFR. Residue based energy decomposition analysis suggested that GLN209 might be a key residue to maintain binding (Figures 5A,B). To test whether cynaropicrin directly bind to LIFR complex, binding profiles of cynaropicrin to LIFR were evaluated using SPR. The results showed cynaropicrin binding to LIFR (Figure 5C). We further verified the effect of cynaropicrin on the STAT3 upstream signaling pathway by demonstrating that the binding capacity of LIFR to STAT3 was inhibited after cynaropicrin treatment through protein immunoprecipitation (Figure 5D). Western blotting was used to detect the LIFR protein levels. The results are shown in Figures 4A,B. The inhibition of LIFR protein level by cynaropicrin treatment was enhanced in a dose- and time-dependent manner. Moreover, LIFR level was reduced prior to STAT3 phosphorylation (Figure 4B). In conclusion, we suggest that cynaropicrin inhibited LIFR level, thereby blocking the downstream activation of the STAT protein.

### Cynaropicrin Stimulation Inhibited the Formation of STAT3-STAT4 Heterodimers

After activation by LIF/LIFR, STAT3 can not only be activated to form a homodimer, but also to form a heterodimer with STAT4 (Zhang et al., 2019). We assessed the influence of cynaropicrin on the formation of STAT3 dimer, and the results of Co-Immunoprecipitation (Co-IP) using antibodies directed against STAT3 and STAT4 are show in Figures 5D,E. The changes in protein bands strongly suggested that the formation of the STAT3/STAT4 heterodimer was inhibited by cynaropicrin. After drug treatment, the binding of STAT3 to LIFR was also negative (Figure 5D). Cellular immunofluorescence assay also demonstrated that STAT4 failed to translocate into the nucleus after cynaropicrin treatment (Figure 5F), which was consistent with the finding that STAT3 translocation into the nucleus was blocked. Therefore, it can be basically concluded that cynaropicrin inhibited LIFR recruitment of STAT3/4 and further reduced STAT3/STAT4 heterodimer formation.

### Cynaropicrin Inhibited the Growth of CRC Xenograft Models

**FIGURE 6:**
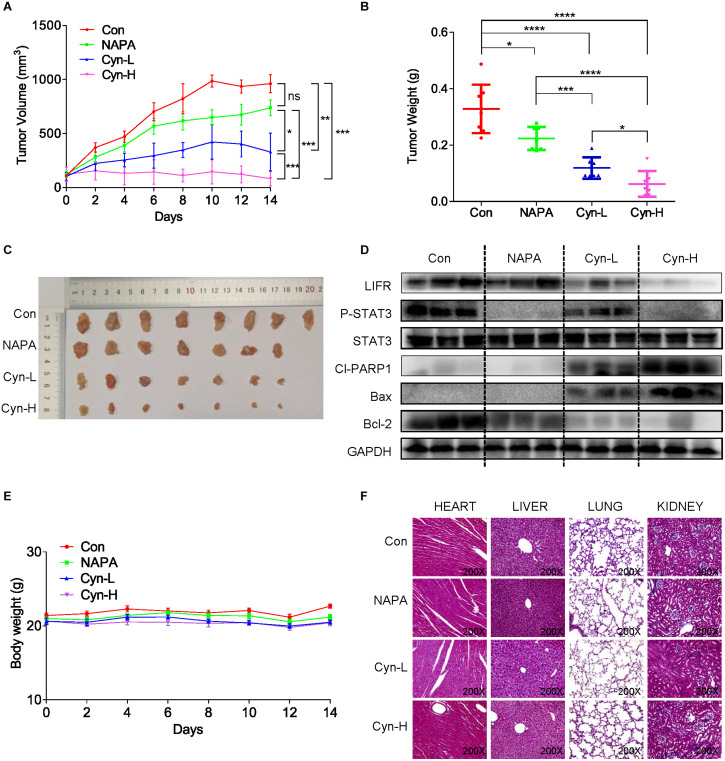
Function of cynaropicrin in tumor xenograft growth inhibition of CRC *in vivo*. **(A)** BALB/c mice were grouped into Control, Napabucasin 10 mg/kg, cynaropicrin 2.5 mg/kg, and cynaropicrin 5 mg/kg. Tumor volumes were recorded every 2 days and the curve was finally plotted. **(B)** At the end of study mice were sacrificed, and the tumors were removed and weighed. **(C)** Gross view of xenograft tumor tissue **(D)** The expression levels of STAT3-related pathway proteins (LIFR, LIF, P-STAT3, and STAT3) and apoptotic proteins (Cl-PARP1, Bax, and Bcl2) in transplanted tumor tissues were detected by Western Blotting. **(E)** The mice were weighed throughout the experiment. **(F)** No histological abnormalities were observed in H&E staining of heart, liver, lung and kidney in the four mice groups. All potency values are expressed as mean ± SD of independent experiments in triplicate. Definition of statistical significance: *P* < 0.05.

BALB/c nude mice were inoculated subcutaneously with HCT116 cells and used as xenograft models to evaluate whether cynaropicrin can inhibit the growth of cancer cells *in vivo*. The STAT3 inhibitor Napabucasin was used as the positive control. Our results showed that intraperitoneal administration of cynaropicrin at doses of 2.5 and 5 mg/kg resulted in decreased tumor volume and weight compared to the vehicle group and Napabucasin (10 mg/kg) group (Figures 6A–C). We found that LIFR and STAT3 phosphorylation was mechanistically inhibited in the treatment groups. Drug treatment also markedly increased apoptosis as indicated by the expression levels of Bax and Bcl-2 (Figure 6D). Moreover, no significant loss of body weight occurred in any of the treatment groups (Figure 6E). We also evaluated the toxicity of cynaropicrin by hematoxylin eosin (H&E) staining analysis in the hearts, livers, lungs and kidneys of mice. No obvious cellular inflammatory, edema or necrosis was observed, demonstrating an excellent safety profile (Figure 6F). These results showed that cynaropicrin had potent antitumor activity against the growth of implanted CRC with minimal toxicity in the animal.

## Discussion

**FIGURE 7:**
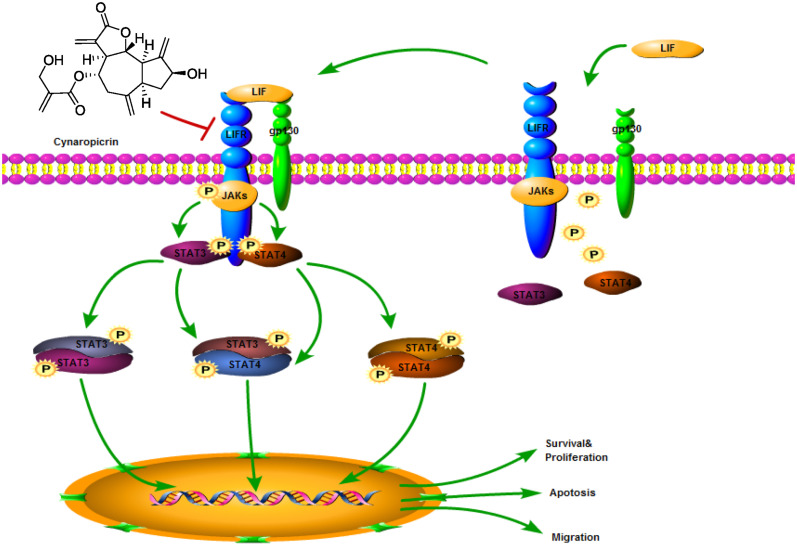
Schematic diagram of the antitumor mechanism of cynaropicrin in CRC by blocking STATs activation into the nucleus by targeting LIFR.

STAT3 has important biological significance and is a potential therapeutic target for CRC (Zhao et al., 2016; Yang et al., 2019). Clinical studies in 724 patients with stage I–IV CRCs showed that STAT3 was significantly associated with poor outcomes and it supported the potential role of STAT3 in pro-tumor inflammatory transmission (Morikawa et al., 2011). Activation of STAT3 has been shown to drive downstream gene transcription, and its gene products subsequently promote tumor development and progression (Du et al., 2012). Our study put forward for the first time that cynaropicrin can inhibit the proliferation of CRC and induce its apoptosis by targeting the LIFR/STATs axis *in vitro* and *in vivo* (Figure 7), suggesting that cynaropicrin is a potential natural product with anti-tumor efficacy.

In recent years, LIFR has been found to play various roles in tumors. As an oncogene, LIFR contributes to the subsequent activation of STAT3 and AKT pathways in prostate cancer, inducing the expression of a series of proliferation-related and transfer genes (Shao et al., 2019; Zhang et al., 2020). In breast cancer, LIFR is a tumor suppressor and metastasis suppressor gene. Abnormal LIFR promoter methylation and down-regulation of protein expression occur, which induce migration, invasion, and metastatic colonization (Chen et al., 2012; Real et al., 2019). As described above, it is very important to identify the role of LIFR in target tumors. According to the existing studies and analyses, LIFR is highly expressed in CRC tumor tissues, which is related to the overall 5-year survival rate after surgery and plays the role of a carcinogen (Wu et al., 2018). Moreover, LIFR reflects the chemosensitivity of metastatic CRC to cetuximab, which may be used to predict the susceptibility of individuals to cetuximab chemotherapy (Kim et al., 2011, 2013). Therefore, it is important to identify drugs that target LIFR in the treatment of CRC. Our results support the claim that cynaropicrin targeted LIFR to block downstream activation of the STATs protein; thereby blocking CRC progression. We must admit that the specific mechanism by which cynaropicrin targets LIFR still has room for further research. Nevertheless, our results are sufficient to indicate that cynaropicrin is a compound with therapeutic potential for CRC.

STAT signals are involved in immune function, cell growth, differentiation, hematopoietic and human cancer development, progression, metastasis, survival, and treatment resistance (Johnson et al., 2018). Based on the results of the present studies, STAT3 may play a relatively important role in tumor and inflammatory biology, while STAT4 appears to be less involved (Verhoeven et al., 2020). However, their role is due to transcriptional modifications controlled by the subtle and complex interactions of different STAT molecules. In the inflammatory pathways of fibroblasts, LIFR forms a molecular complex with STAT4, which initiates STAT4 activation. STAT4 then enters the nucleus and is recruited to the IL-6 promoter (Nguyen et al., 2017). A positive feedback loop involving LIF, LIFR, and STAT4 drives sustained IL-6 transcription. In laminar propria lymphocytes of inflammatory bowel disease patients, LIF-activated STAT4 inhibits activation of the STAT3-dependent Il17a/Il17f promoter, while in intestinal epithelial cells, LIF bypasses abnormally low STAT4 levels and induces YAP gene expression by activating STAT3 (Zhang et al., 2019). Therefore, our attempt to investigate the interaction between STAT3 and STAT4 proteins in CRC was meaningful. LIF stimulation can induce LIFR to recruit STAT3 and STAT4. Our experimental data demonstrated that cynaropicrin inhibited the formation of this complex and blocked the formation of STAT3-4 heterodimer. LIFR protein was positive when immunoprecipitation was performed using STAT3 primary antibody but negative with STAT4 primary antibody. We consider that it is probably a consequence of insufficient STAT4 protein abundance.

Cynaropicrin is one of the main active ingredients of artichoke. Maximum tolerated dose (MTD) was assessed in Swiss–Webster female rats for acute toxicity testing. The results showed that MTD for an intraperitoneal injection was 200 mg/kg and for oral administration was 400 mg/kg, which was much higher than the IC50 value measured in CRC cells in our study (da Silva et al., 2013). Therefore, the inference that cynaropicrin is safe and less toxic is basically reliable. In addition to its own anti-tumor efficacy, cynaropicrin increased the chemotherapeutic sensitivity of tumor cells, showing a mild to strong synergistic effect with cisplatin and docetaxel (Butturini et al., 2013). As a natural product, it has obvious advantages if it can be an effective therapeutic drug. On the one hand, the compound is water-soluble and can therefore be configured with therapeutic injections to shorten the onset time and thus reduce the possible side effects (de Faria et al., 2017). On the other hand, its simple structure and low cost of synthesis and manufacturing can reduce the medical treatment burden of patients (Elsebai et al., 2016b).

In summary, our study suggested that cynaropicrin causes potential inhibition of CRC *in vitro* and *in vivo* by suppressing the activation of LIFR/STATs signaling pathway. Our findings, together with the work of our predecessors, reveal the potential therapeutic value of cynaropicrin. However, further strategies need to be elucidated to optimize the clinical use of cynaropicrin.

## Data Availability Statement

The original contributions presented in the study are included in the article/Supplementary Material and further inquiries can be directed to the corresponding author/s.

## Ethics Statement

The animal study was reviewed and approved by the Institutional Animal Care and Use Committee of Wenzhou Medical University.

## Author Contributions

DDZ, YZ, and YLS carried out most of the experiments. SSX, LHY, YQX, XXD, WLH, BZ, and ZGL participated in part of the experiment and analyzed the data. HYZ prerevised the manuscript. CGZ, XYH, and LXW conceived the idea and designed the research. CGZ, XYH, and DDZ wrote the manuscript. All authors read and approved final version of the manuscript.

## Conflict of Interest

The authors declare that the research was conducted in the absence of any commercial or financial relationships that could be construed as a potential conflict of interest.
