# Supplementary material for: Cynaropicrin Shows Antitumor Progression Potential in Colorectal Cancer Through Mediation of the LIFR/STATs Axis
Source: Front Cell Dev Biol. 2021 Jan 11;8:605184. doi: 10.3389/fcell.2020.605184 (PMC7829511; doi:10.3389/fcell.2020.605184)
Supplement: Supplementary file 1 [file Presentation_1.PPTX]

## Slide 1
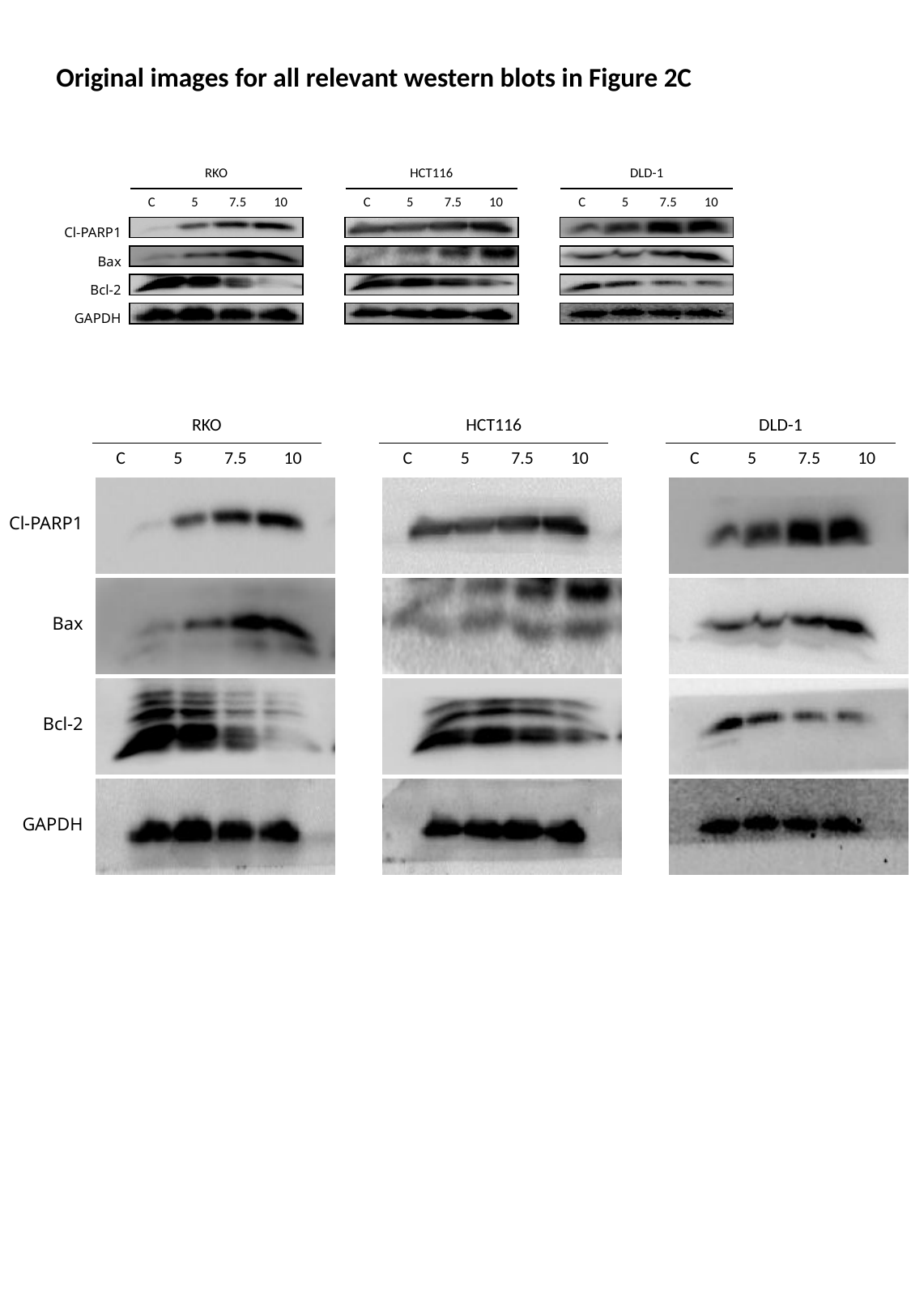

Original images for all relevant western blots in Figure 2C
| RKO | | | | | HCT116 | | | | | DLD-1 | | | |
| --- | --- | --- | --- | --- | --- | --- | --- | --- | --- | --- | --- | --- | --- |
| C | 5 | 7.5 | 10 | | C | 5 | 7.5 | 10 | | C | 5 | 7.5 | 10 |
| Cl-PARP1 |
| --- |
| Bax |
| Bcl-2 |
| GAPDH |
| RKO | | | | | HCT116 | | | | | DLD-1 | | | |
| --- | --- | --- | --- | --- | --- | --- | --- | --- | --- | --- | --- | --- | --- |
| C | 5 | 7.5 | 10 | | C | 5 | 7.5 | 10 | | C | 5 | 7.5 | 10 |
| Cl-PARP1 |
| --- |
| Bax |
| Bcl-2 |
| GAPDH |

## Slide 2
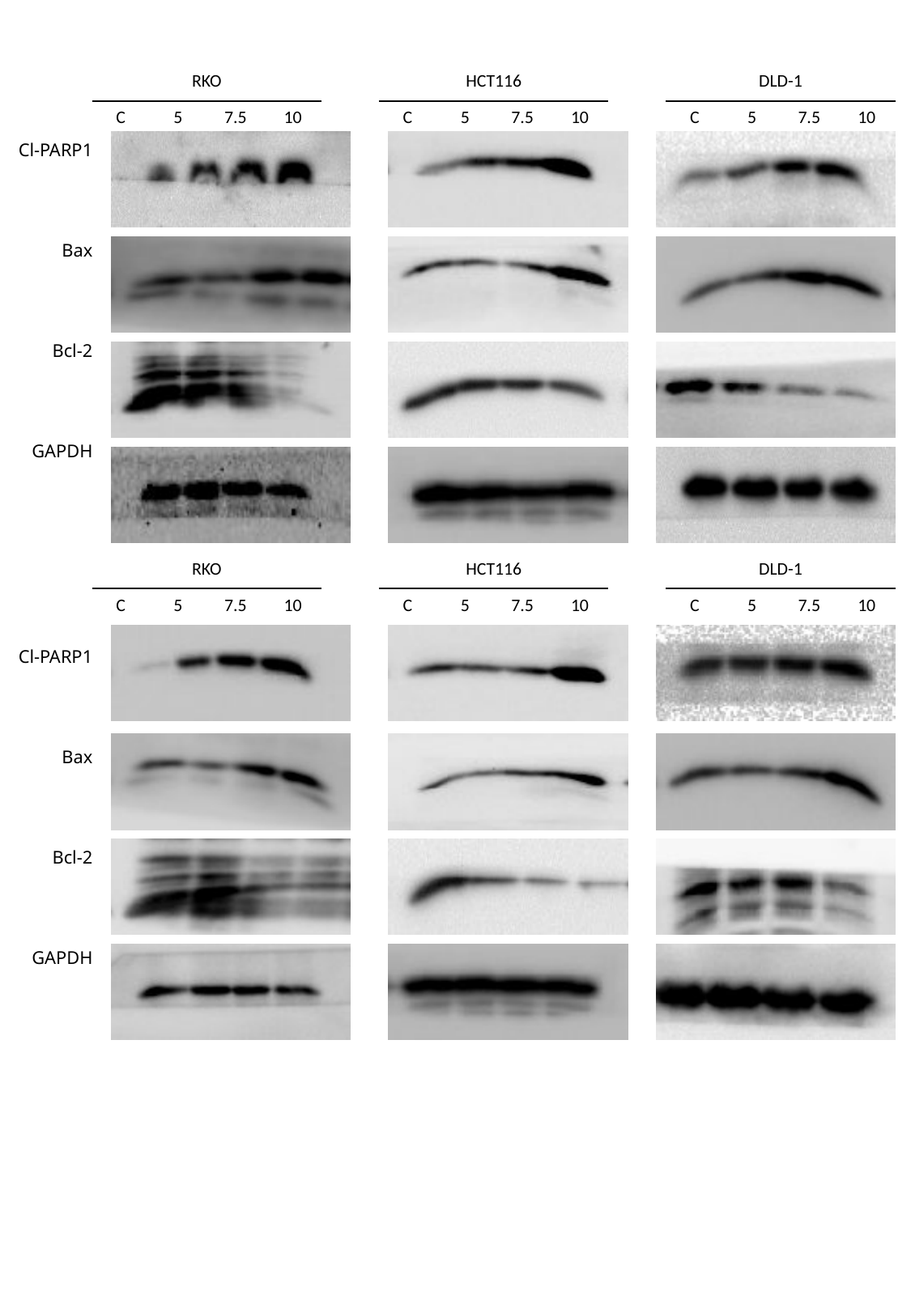

| RKO | | | | | HCT116 | | | | | DLD-1 | | | |
| --- | --- | --- | --- | --- | --- | --- | --- | --- | --- | --- | --- | --- | --- |
| C | 5 | 7.5 | 10 | | C | 5 | 7.5 | 10 | | C | 5 | 7.5 | 10 |
| Cl-PARP1 |
| --- |
| Bax |
| Bcl-2 |
| GAPDH |
| RKO | | | | | HCT116 | | | | | DLD-1 | | | |
| --- | --- | --- | --- | --- | --- | --- | --- | --- | --- | --- | --- | --- | --- |
| C | 5 | 7.5 | 10 | | C | 5 | 7.5 | 10 | | C | 5 | 7.5 | 10 |
| Cl-PARP1 |
| --- |
| Bax |
| Bcl-2 |
| GAPDH |

## Slide 3
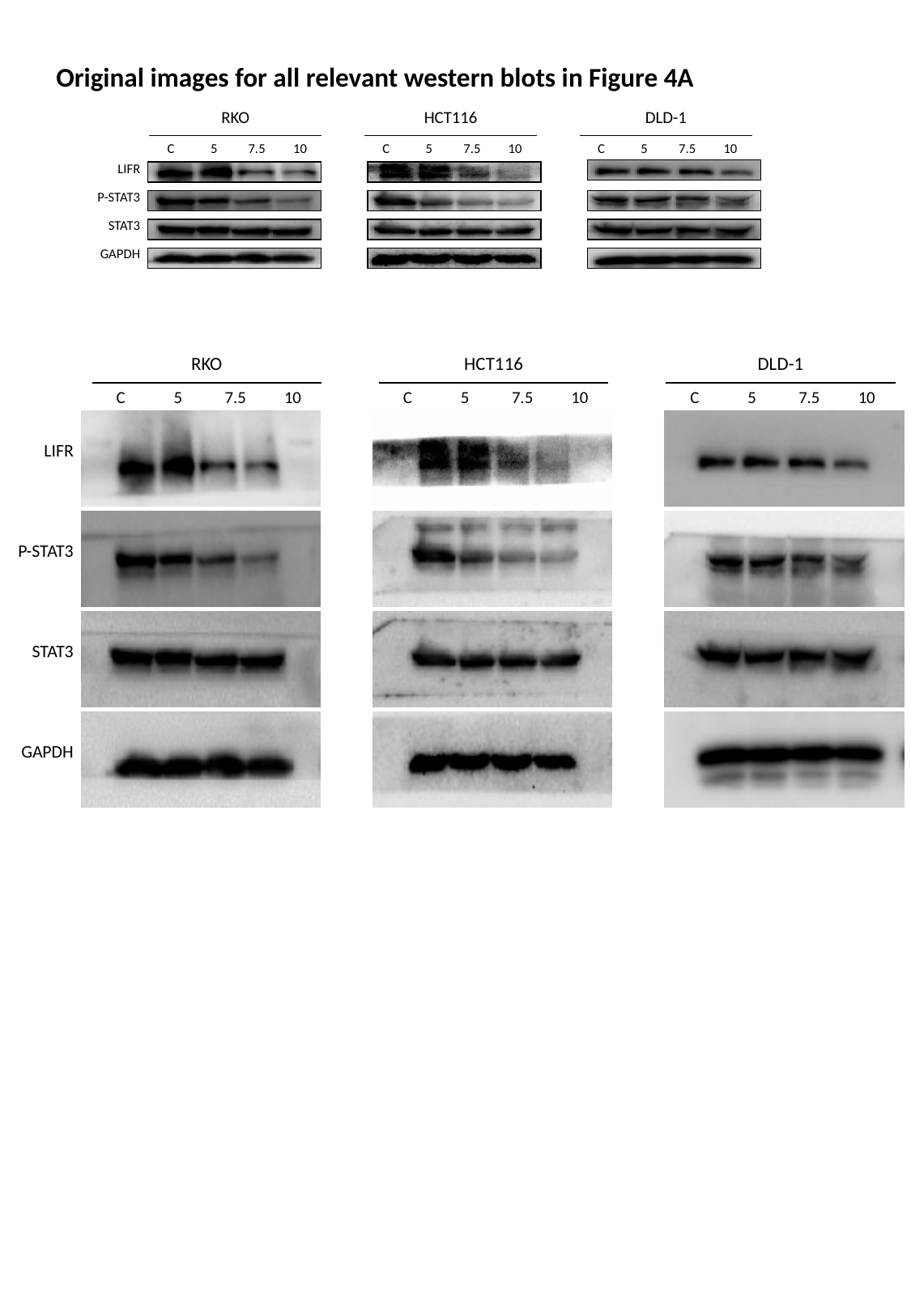

Original images for all relevant western blots in Figure 4A
| RKO | | | | | HCT116 | | | | | DLD-1 | | | |
| --- | --- | --- | --- | --- | --- | --- | --- | --- | --- | --- | --- | --- | --- |
| C | 5 | 7.5 | 10 | | C | 5 | 7.5 | 10 | | C | 5 | 7.5 | 10 |
| LIFR |
| --- |
| P-STAT3 |
| STAT3 |
| GAPDH |
| RKO | | | | | HCT116 | | | | | DLD-1 | | | |
| --- | --- | --- | --- | --- | --- | --- | --- | --- | --- | --- | --- | --- | --- |
| C | 5 | 7.5 | 10 | | C | 5 | 7.5 | 10 | | C | 5 | 7.5 | 10 |
| LIFR |
| --- |
| P-STAT3 |
| STAT3 |
| GAPDH |

## Slide 4
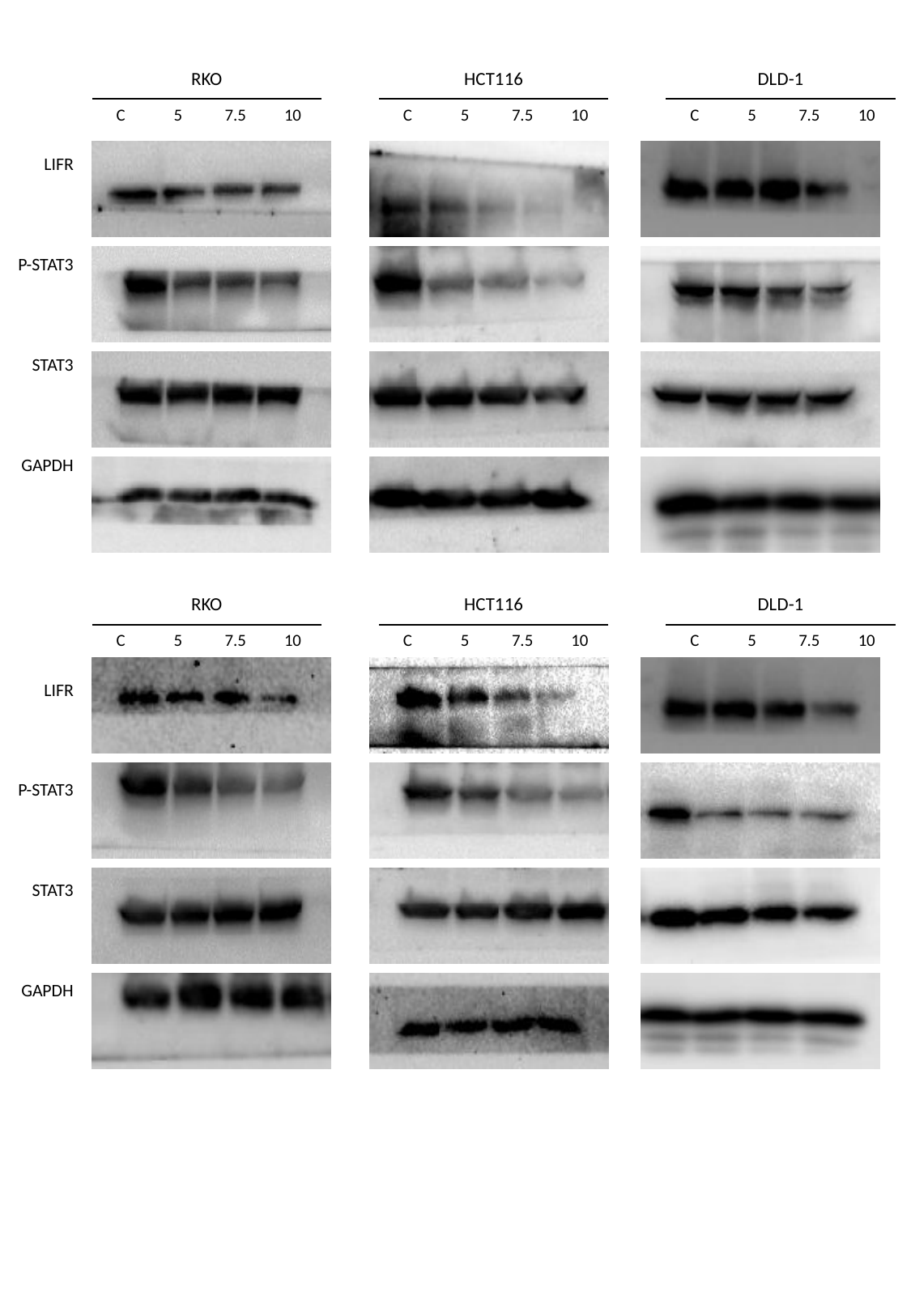

| RKO | | | | | HCT116 | | | | | DLD-1 | | | |
| --- | --- | --- | --- | --- | --- | --- | --- | --- | --- | --- | --- | --- | --- |
| C | 5 | 7.5 | 10 | | C | 5 | 7.5 | 10 | | C | 5 | 7.5 | 10 |
| LIFR |
| --- |
| P-STAT3 |
| STAT3 |
| GAPDH |
| RKO | | | | | HCT116 | | | | | DLD-1 | | | |
| --- | --- | --- | --- | --- | --- | --- | --- | --- | --- | --- | --- | --- | --- |
| C | 5 | 7.5 | 10 | | C | 5 | 7.5 | 10 | | C | 5 | 7.5 | 10 |
| LIFR |
| --- |
| P-STAT3 |
| STAT3 |
| GAPDH |

## Slide 5
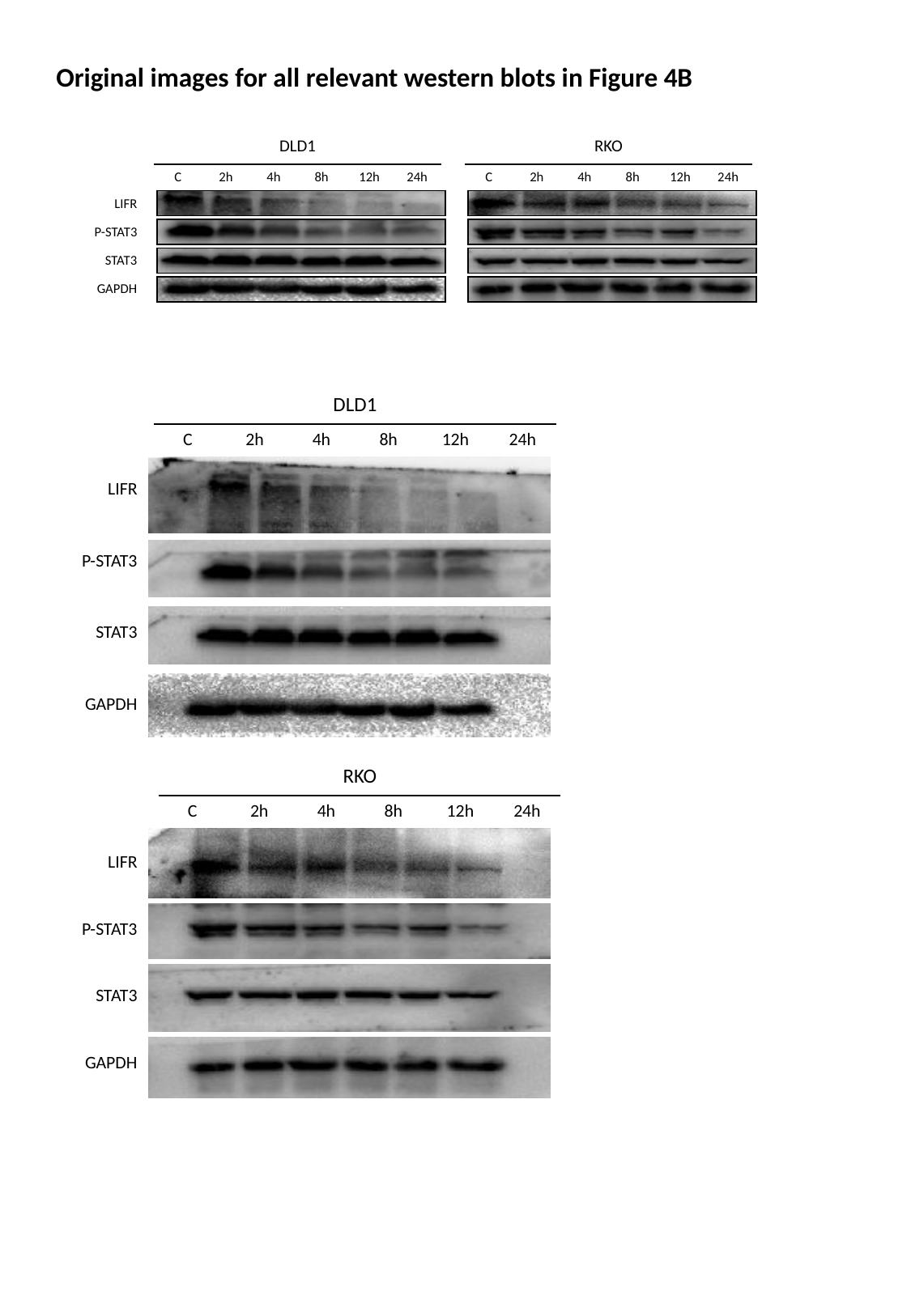

Original images for all relevant western blots in Figure 4B
| DLD1 | | | | | |
| --- | --- | --- | --- | --- | --- |
| C | 2h | 4h | 8h | 12h | 24h |
| RKO | | | | | |
| --- | --- | --- | --- | --- | --- |
| C | 2h | 4h | 8h | 12h | 24h |
| LIFR |
| --- |
| P-STAT3 |
| STAT3 |
| GAPDH |
| DLD1 | | | | | |
| --- | --- | --- | --- | --- | --- |
| C | 2h | 4h | 8h | 12h | 24h |
| LIFR |
| --- |
| P-STAT3 |
| STAT3 |
| GAPDH |
| RKO | | | | | |
| --- | --- | --- | --- | --- | --- |
| C | 2h | 4h | 8h | 12h | 24h |
| LIFR |
| --- |
| P-STAT3 |
| STAT3 |
| GAPDH |

## Slide 6
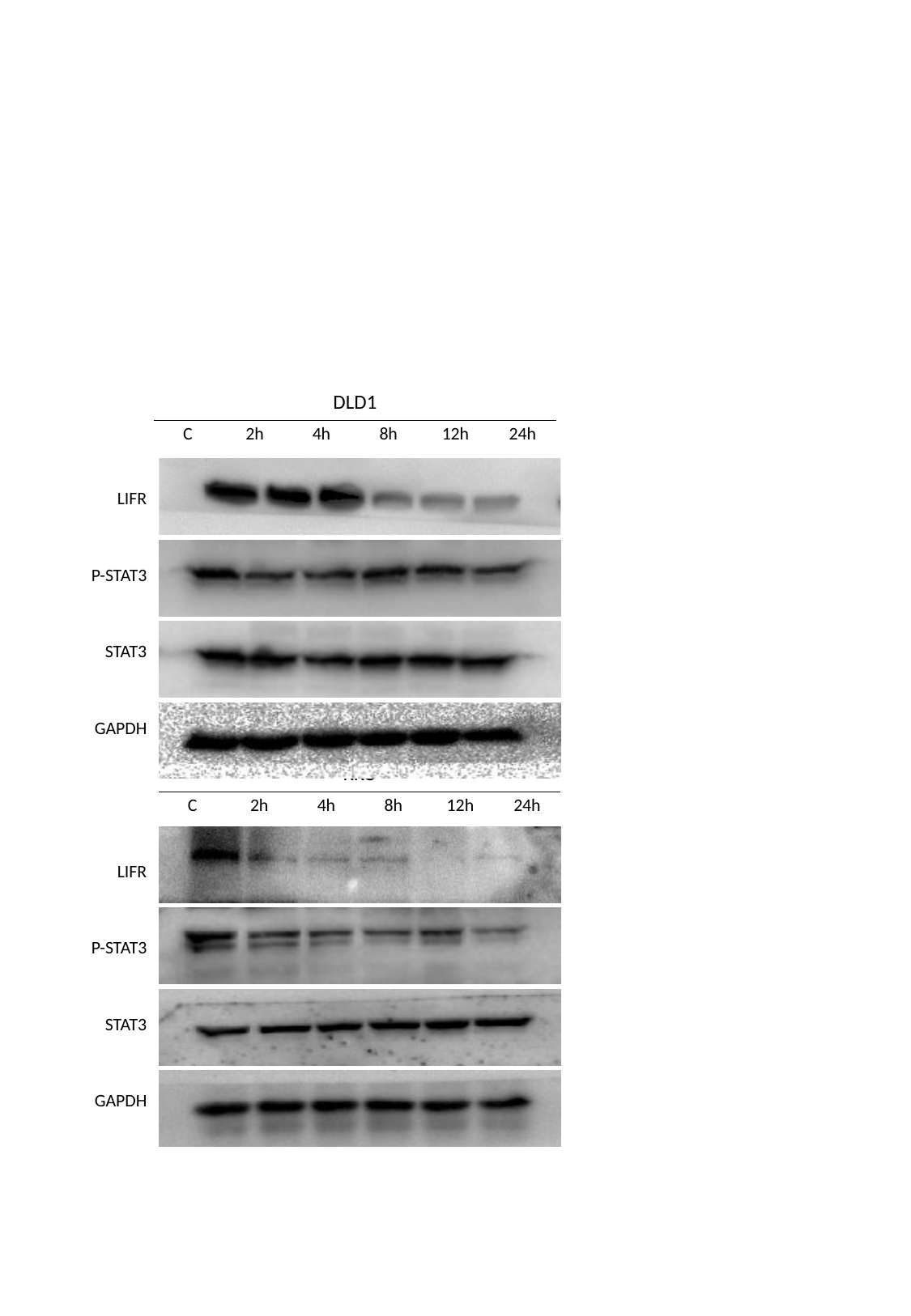

| DLD1 | | | | | |
| --- | --- | --- | --- | --- | --- |
| C | 2h | 4h | 8h | 12h | 24h |
| LIFR |
| --- |
| P-STAT3 |
| STAT3 |
| GAPDH |
| RKO | | | | | |
| --- | --- | --- | --- | --- | --- |
| C | 2h | 4h | 8h | 12h | 24h |
| LIFR |
| --- |
| P-STAT3 |
| STAT3 |
| GAPDH |

## Slide 7
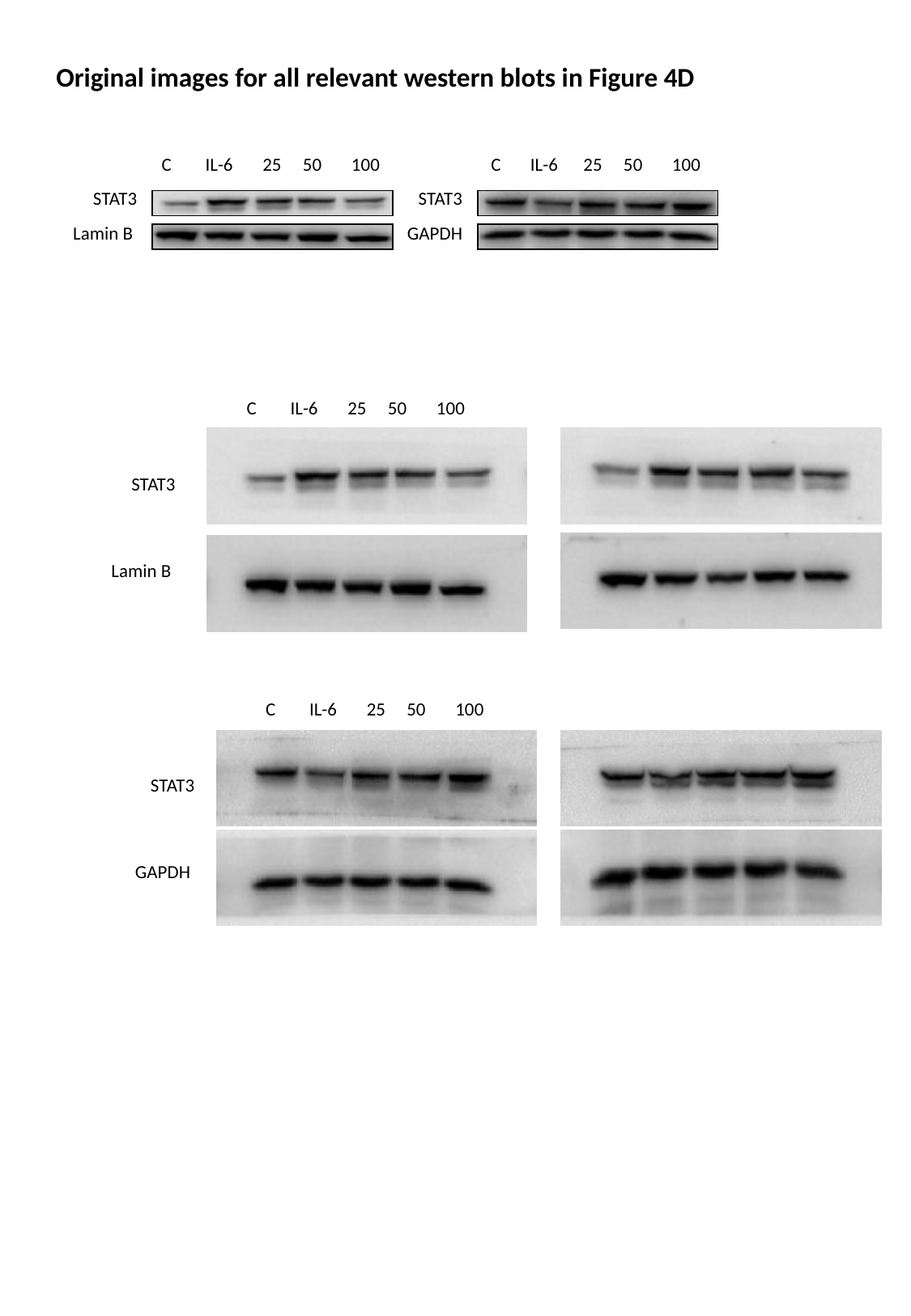

Original images for all relevant western blots in Figure 4D
| | C IL-6 25 50 100 |
| --- | --- |
| STAT3 | |
| Lamin B | |
| | C IL-6 25 50 100 |
| --- | --- |
| STAT3 | |
| GAPDH | |
| | C IL-6 25 50 100 |
| --- | --- |
| STAT3 | |
| Lamin B | |
| | C IL-6 25 50 100 |
| --- | --- |
| STAT3 | |
| GAPDH | |

## Slide 8
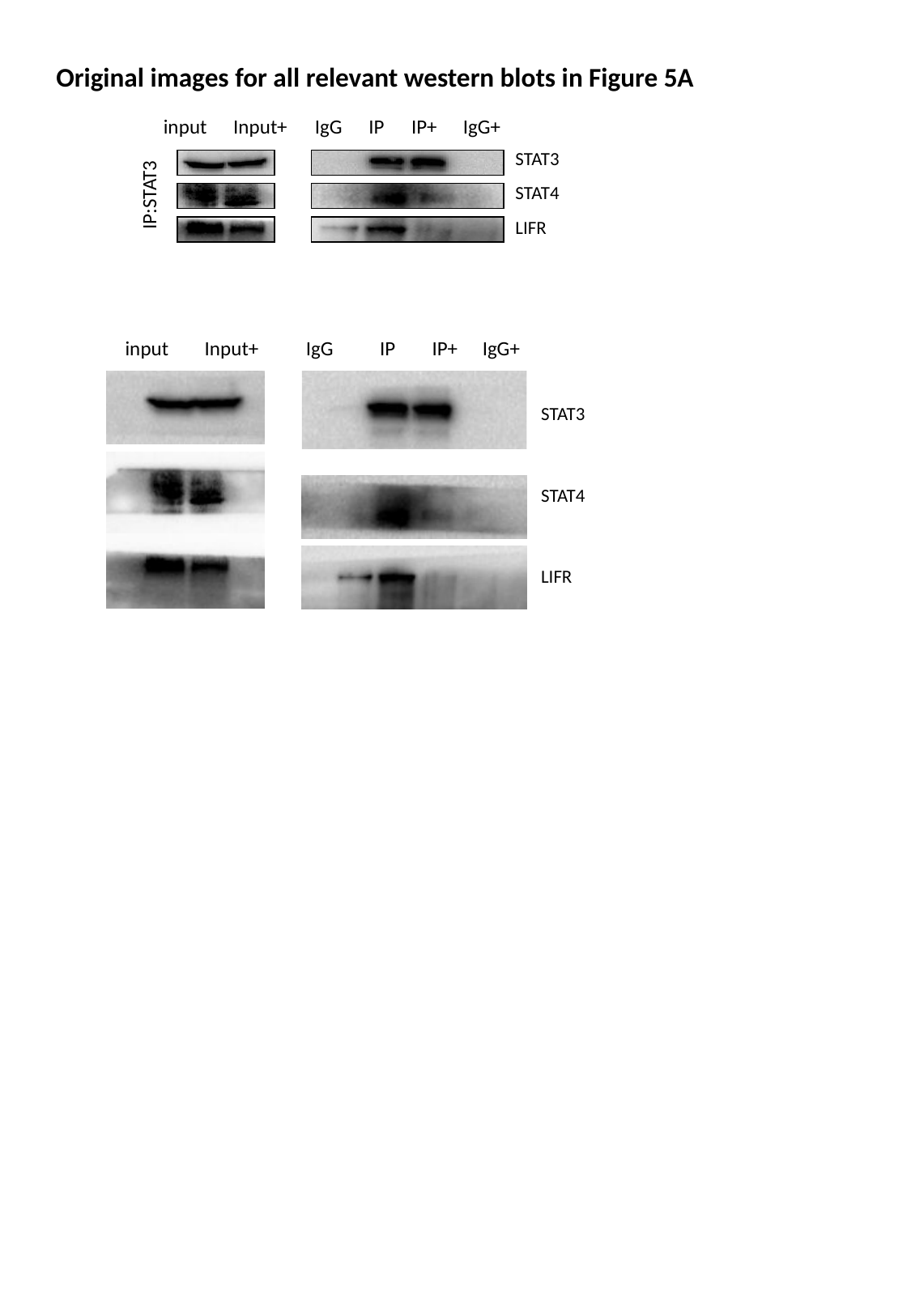

Original images for all relevant western blots in Figure 5A
| input | Input+ | IgG | IP | IP+ | IgG+ |
| --- | --- | --- | --- | --- | --- |
| STAT3 |
| --- |
| STAT4 |
| LIFR |
IP:STAT3
| input | Input+ | IgG | IP | IP+ | IgG+ |
| --- | --- | --- | --- | --- | --- |
| STAT3 |
| --- |
| STAT4 |
| LIFR |

## Slide 9
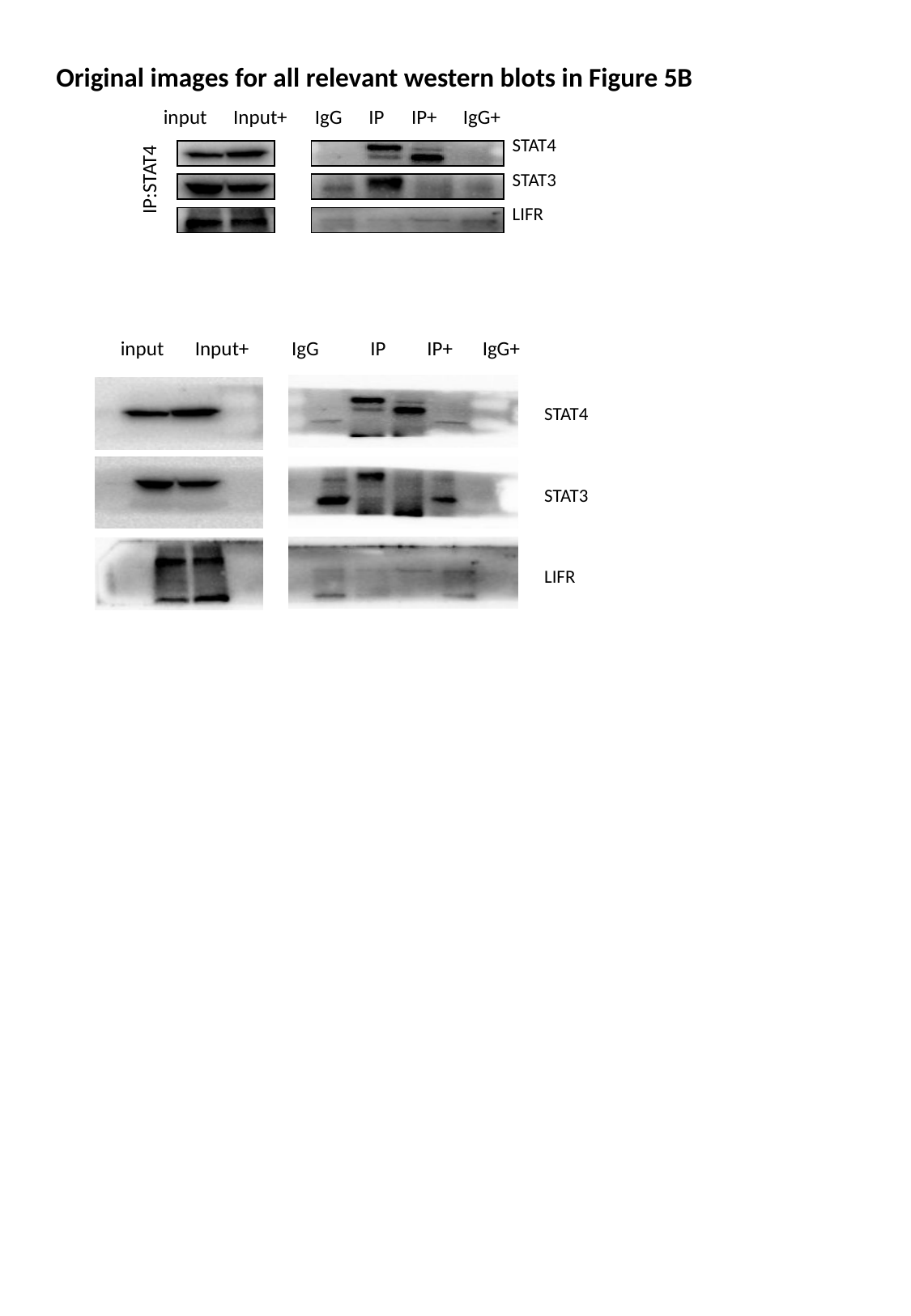

Original images for all relevant western blots in Figure 5B
| input | Input+ | IgG | IP | IP+ | IgG+ |
| --- | --- | --- | --- | --- | --- |
| STAT4 |
| --- |
| STAT3 |
| LIFR |
IP:STAT4
| input | Input+ | IgG | IP | IP+ | IgG+ |
| --- | --- | --- | --- | --- | --- |
| STAT4 |
| --- |
| STAT3 |
| LIFR |

## Slide 10
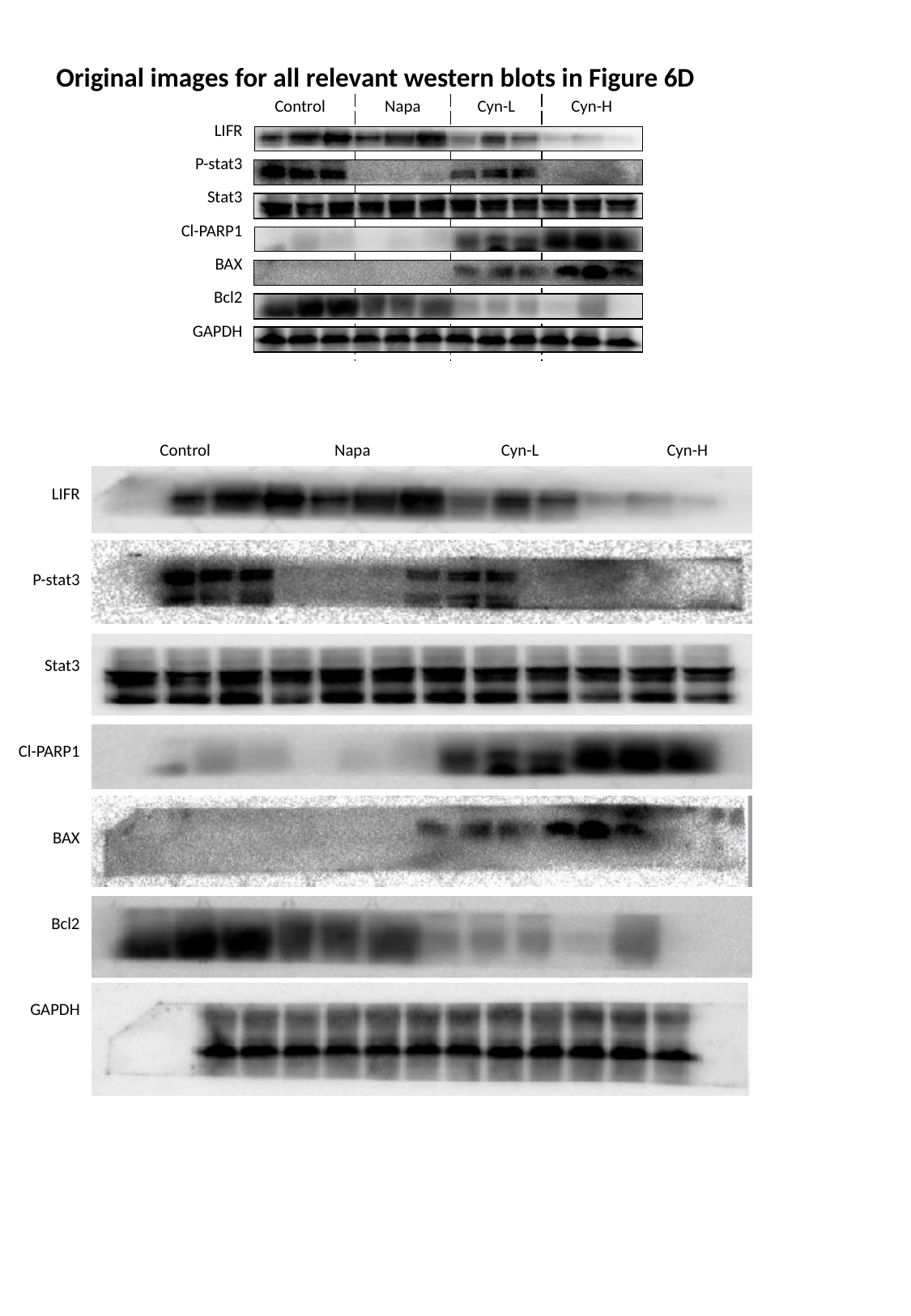

Original images for all relevant western blots in Figure 6D
| Control | Napa | Cyn-L | Cyn-H |
| --- | --- | --- | --- |
| | | | |
| | | | |
| | | | |
| | | | |
| | | | |
| | | | |
| LIFR |
| --- |
| P-stat3 |
| Stat3 |
| Cl-PARP1 |
| BAX |
| Bcl2 |
| GAPDH |
| Control | Napa | Cyn-L | Cyn-H |
| --- | --- | --- | --- |
| | | | |
| | | | |
| | | | |
| | | | |
| | | | |
| | | | |
| LIFR |
| --- |
| P-stat3 |
| Stat3 |
| Cl-PARP1 |
| BAX |
| Bcl2 |
| GAPDH |
