# Supplementary figures and images for: Cynaropicrin Shows Antitumor Progression Potential in Colorectal Cancer Through Mediation of the LIFR/STATs Axis
Source: Front Cell Dev Biol. 2021 Jan 11;8:605184. doi: 10.3389/fcell.2020.605184 (PMC7829511; doi:10.3389/fcell.2020.605184)

## Slide 1
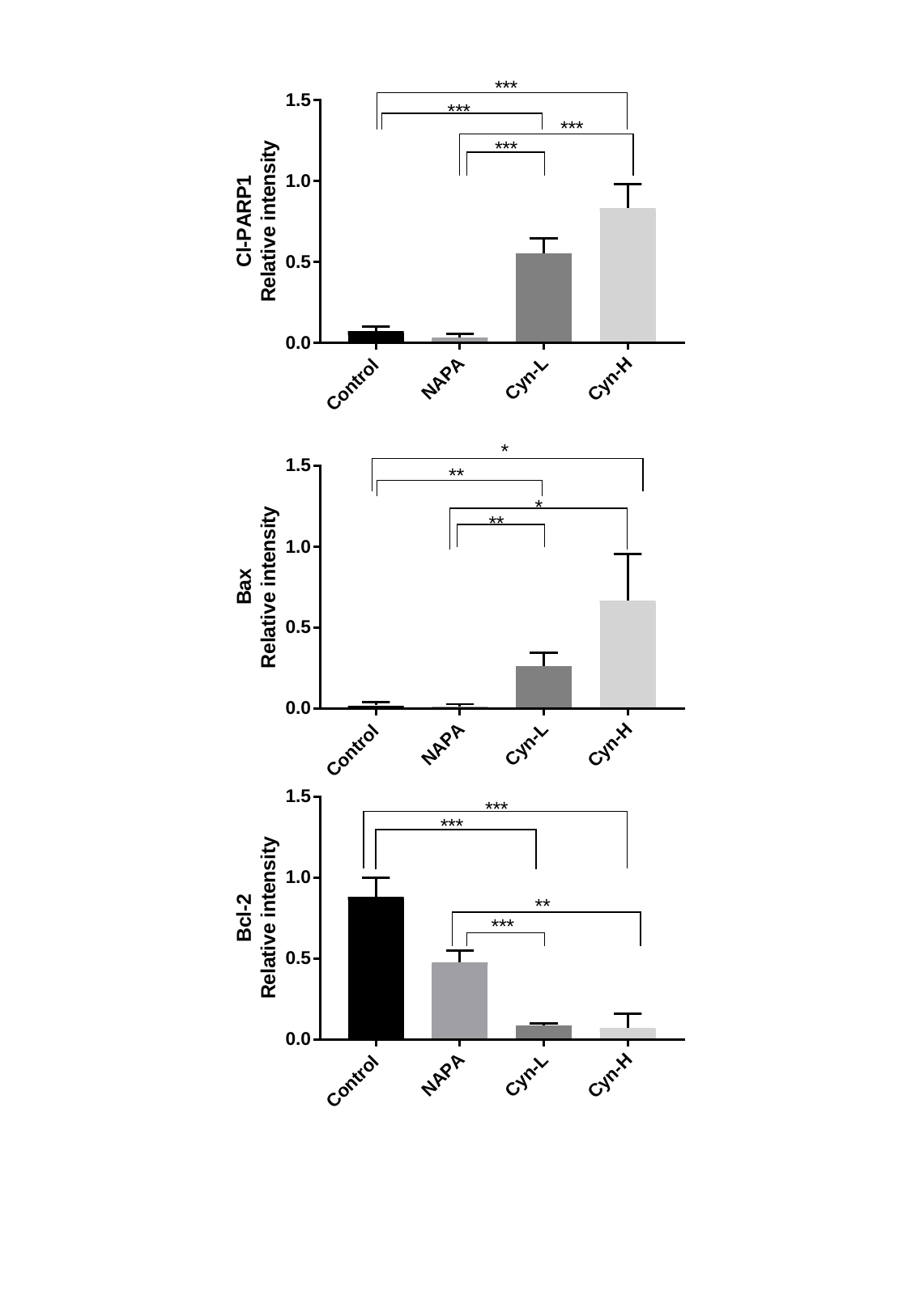

Supplement: Supplementary file 2 [file Presentation_2.PPTX]
